# Targeted Poverty Alleviation and Households’ Livelihood Strategy in a Relation-Based Society: Evidence from Northeast China

**DOI:** 10.3390/ijerph18041747

**Published:** 2021-02-11

**Authors:** Zhe Sun, Liang Zhao, Shuyue Wang, Hongyin Zhang, Xinyu Wang, Zherui Wan

**Affiliations:** 1 Center for China Public Sector Economy Research, Economics School, Jilin University, Changchun 130012, China; sunzhe@jlu.edu.cn (Z.S.); wangsy0617@mails.jlu.edu.cn (S.W.); zhanghy0617@mails.jlu.edu.cn (H.Z.); wangxy0617@mails.jlu.edu.cn (X.W.); wanzr0617@mails.jlu.edu.cn (Z.W.); 2 School of Business and Law, University of Agder, 4630 Kristiansand, Norway

**Keywords:** relation-based society, livelihood strategy, grounded theory, targeted poverty alleviation

## Abstract

Although China is experiencing a transition from a relation-based society to a rule-based society, relationships among acquaintances still play an important role in resource allocation, such as the allocation of policy resources. This is particularly true in rural China, where targeted poverty alleviation is prevalent and a relation-based social structure still dominates. However, it is still unknown how relationships affect the livelihood strategy of households in rural China and how poverty alleviation policies plays a role between them. Therefore, this paper embeds poverty alleviation into the relation-based society and explores how households respond to the policy in this specific context. Using grounded theory research method and the sustainable livelihoods approach (SLA) framework, this paper contains in-depth interviews and field observations from three poverty-stricken villages in Northeast China. The results show that relationships have a significant impact on the households’ livelihood strategy. In other words, the households’ livelihood strategy is embedded in the relation-based society. The types of relationships induce households to choose maintained or developmental type livelihood strategies, while relationships influence how the poverty alleviation policies affect the livelihood strategy. This study is not only an extension of the SLA in the research context, but also provides a significant perspective for enriching the long-term mechanism of targeted poverty alleviation by building a theoretical model of the relationships between a relation-based society, targeted poverty alleviation and the livelihood strategies of households.

## 1. Introduction

Poverty is a global problem facing the world today. As the largest developing country in the world, China has explored an anti-poverty route with Chinese characteristics in the long-term practice of poverty alleviation [1]. Remarkably, China has achieved a leap from universal poverty to a general well-off state, which has made great contributions to the world’s poverty reduction [2]. In 2013, the central leadership led by Chairman Xi Jinping put forward the idea of “helping the poor with precision”, which is also termed a targeted poverty alleviation policy, calling on local governments to make better use of poverty alleviation resources and take targeted measures to ensure that assistance reaches poverty-stricken villages and households [3]. The rural poverty-stricken population decreased from 98.99 million in 2013 to 16.6 million in 2018, with a cumulative reduction of 82.39 million, whereas the scale of poverty reduction reached more than 10 million every year for six consecutive years, and the poverty incidence rate dropped from 10.2% to 1.7% [4].

Despite these great achievements, establishment of a long-term mechanism for poverty alleviation policies remains challenging. The problem is that policy orientation often focuses on the quantitative aspects of poverty alleviation, yet the quality aspect, for instance the livelihood of people, is sometimes neglected [1]. The livelihood strategies people make implement depend on the specific context that people live in [5]. Therefore, the specific context constitutes an important perspective from which to understand the poverty-stricken population and their livelihood strategies. Although the topic has been discussed extensively in the literature in China [6,7,8], the discussion about Northeast China has received little attention.

It is worth noting that China is experiencing a transition from a relation-based society to a rule-based society [9], but relation-based governance remains dominant in the rural areas of Northeast China. Relationships among acquaintances formed by kinship and geographical relations still play an important role in resource allocation [6,7,8]. In the actual process of policy alleviation, some practices largely neglect the reality of relation-based social structure in rural areas and the phenomenon of helping the poor through relationships occurs frequently [10]. The poverty alleviation resources are often distributed to households who have relationships instead of truly poverty-stricken ones [11]. A direct consequence is the inefficient distribution of resources and the low efficiency of poverty alleviation policies. The consequence is particularly serious in the current targeted poverty alleviation that requires poverty alleviation resources to be precisely delivered to households.

With the aim to developing an efficient way of alleviating poverty in a sustainable sense, it is imperative to embed the poverty alleviation practices into the specific context of rural areas in China. The sustainable livelihood approach (SLA) provides a useful analytical framework for this purpose [12]. Taking contextual factors into consideration, the SLA argues that households’ livelihood strategy is not only a way of combination and use of their own livelihood assets, but also a response to existing contextual factors [13]. Therefore, it might be appropriate to adopt the SLA to investigate how households choose a livelihood strategy, and how they respond to targeted poverty alleviation policies in the specific context of a relation-based society.

Based on the SLA, this paper explores the role of the specific context of relation-based society and targeted poverty alleviation policies in households’ choice of livelihood strategies in rural areas of Northeast China. In theory, this paper attempts to build a theoretical model of the relationship between a relation-based society, targeted poverty alleviation policies and the livelihood strategies of households. Thus, this study is not only an extension of the SLA in the research context, but also provides a significant perspective for enriching the long-term mechanism of targeted poverty alleviation. In practice, this paper aims to refine the corresponding countermeasures and suggestions from the perspective of both households and the government. In so doing, the paper attempts to provide new ideas for households to achieve sustainable livelihoods and for government departments to formulate effective long-term poverty alleviation policies.

## 2. Evolution of Poverty Alleviation Policy in China

The Chinese government has been dedicated to antipoverty efforts for the past decades [14]. In the early days of the founding of the new China, poverty was a widespread common phenomenon. The poverty alleviation work has been gradually developed on the basis of rural relief [15]. The top-down, large-scale and nationwide poverty alleviation work was formally proposed and implemented after the reform and opening up in 1978 [14]. Therefore, this paper reviews China’s poverty alleviation policy since the year 1978, and divides the anti-poverty process into five periods [1,14,15].

### 2.1. The First Period (1978–1985): Institutional Reform Antipoverty

At the end of 1978, the Third Plenary Session of the Eleventh Central Committee of the Communist Party of China (CPC) clearly pointed out that it is necessary to make some people and some regions rich first, and finally achieve common prosperity. The prelude of reform and opening up was thus initiated. Since then, the poverty standard has been formally defined, and the poverty alleviation work has been separated from the rural relief for the first time. The driving force of poverty reduction at that time mainly came from the rural economic reform [15]. In January 1982, the CPC Central Committee approved the summary of the National Rural Work Conference, the main contents of which are household contract responsibility system and agricultural product price adjustment [16]. In September 1984, the State Council issued “the Notice on Helping Poor Areas to Change their Appearance as Soon as Possible”. This is for the first time that the CPC and the central government put forward poverty alleviation as an important task. On this basis, China’s large-scale poverty alleviation work was carried out, and the concept of poverty alleviation has changed from the original relief-type poverty alleviation to take regional development as the goal. However, on the whole, China’s poverty alleviation policy still focused on relief.

### 2.2. The Second Period (1986–1993): Large-Scale Development-Oriented Poverty Alleviation

In April 1986, the Fourth Session of the Sixth National People’s Congress deliberated the Seventh Five-Year Plan for National Economic and Social Development (the Seventh Five-Year Plan in short). It highlighted the economic development of “old, young, border and poor” areas. In the same year, a special poverty alleviation agency, the Leading Group for Economic Development in Poor Areas under the State Council was established (later renamed as the Leading Group for Poverty Alleviation and Development of the State Council). The agency was to draw up the policy of economic development in poverty-stricken areas, to arrange special support funds, and formulate special reform and development policies according to the characteristics of poverty-stricken areas and people. The establishment of the agency has effectively promoted the development and poverty reduction in economically backward areas. As a result, China’s poverty alleviation has gradually entered the stage of large-scale planned and organized development-oriented poverty alleviation. At the end of 1986, China first established the standards for national poverty-stricken counties. In “the Notice on Strengthening Economic Development in Poverty-Stricken Areas” issued by the State Council in 1987, it was mentioned that “the fundamental transformation from simple relief to economic development has been initially completed in the national rural poverty-stricken areas, and has begun to enter a new stage of development.” Afterwards, a series of poverty alleviation policies including work-relief programs and financial support funds were issued [14,15].

### 2.3. The Third Period (1994–2000): Crucial Priority Poverty Alleviation

The promulgation and enforcement of the “National Eight-Seven Poverty Alleviation Program (1994–2000)” in 1994 marked that China’s poverty alleviation has entered a critical stage. The plan decided to concentrate human, physical and financial resources, and strive to basically solve the problem of food and clothing for 80 million rural poverty-stricken people in about seven years from 1994 to 2000 [17]. Meanwhile, the plan incorporated the poverty alleviation in the central and western regions into the comprehensive plan for national economic development. In addition, the standard for poverty-stricken counties has been adjusted, and 592 key poverty-stricken counties have been redefined. Since 1994, social forces of poverty alleviation have gradually grown. Various social organizations, non-governmental organizations and private enterprises have actively carried out poverty alleviation activities, such as “the Hope Project”, “the Glorious Cause Project”, “the Happiness Project”, “the Spring Bud Plan”, “the Relay Plan for Youth Volunteers to Support Education and Poverty Alleviation” and “the Self Supporting Project for Poor Farmers”. At this period, China persisted in the implementation of development-oriented poverty alleviation, continued to adopt regional and county-level targeting measures, and gradually systematized and institutionalized poverty alleviation policies by means of poverty alleviation loans and strengthening social mobilization.

### 2.4. The Fourth Period (2001–2010): Comprehensive Poverty Alleviation

In June 2001, the State Council issued the “Outline of China’s Rural Poverty Alleviation and Development (2001–2010)”. The new policy attempted to build a multidimensional and comprehensive pattern of poverty alleviation and changed the focus of poverty alleviation from the original “county” to a more detailed “village” level [18]. The policy aimed to improving the self-development ability of the rural poor population by developing the planting and breeding industry, promoting the agricultural industrialization management, improving the basic production and living conditions, strengthening the poverty alleviation through science and technology, and improving the cultural quality. Since then, China’s poverty alleviation has entered the stage of comprehensive poverty alleviation. In October 2008, the third session of the 17th CPC Central Committee deliberated and passed the “Decision of the CPC Central Committee on Several Major Issues of Promoting Rural Reform and Development”. The decision proposed new poverty alleviation standards, the comprehensive implementation of poverty alleviation policies for rural low-income population, and the promotion the development of rural culture, education, medical and health and other public utilities. In this stage, with the goal of building a well-off society in an all-round way, China adopted the village-level targeting measures to continuously improve the poverty alleviation policy system.

### 2.5. The Fifth Period (2011-Present): Targeted Poverty Alleviation

The poverty alleviation policy at this stage fully reflects the coming of a new era of poverty alleviation development in China. In 2011, the “Outline of China’s Rural Poverty Alleviation Development (2011–2020)” was released. Differing from the previous poverty alleviation on regional development, the central government pointed out that it is necessary to “establish and improve the identification mechanism of poverty alleviation objects”. Thus, the target of poverty alleviation policy has been focused from “county” and “village” to more accurate “poor households” and “poor population”. In November 2013, when Xi Jinping visited Xiangxi, Hubei, he made the first instruction of “helping the poor with precision” [1]. In January 2014, the central government started to regulate the top-level design of the targeted poverty alleviation, and the idea of “targeted poverty alleviation” began to come into effect. In June 2014, the Poverty Alleviation Office of the State Council issued the “Implementation Plan for Establishing A Targeted Poverty Alleviation Mechanism”, which described in detail the four main contents of targeted poverty alleviation: accurate identification, precise assistance, precise management and accurate assessment [13]. Since then, targeted poverty alleviation has become the basic policy of poverty alleviation, and China has successively issued a number of guidance documents focusing on targeted poverty alleviation.

In summary, the poverty alleviation policies have experienced a transition from relief focused to development centered, the way of poverty alleviation has changed from the government led to the participation of all sectors in the society. In particular, unlike the previous poverty alleviation policies, the targeted poverty alleviation policy is more people-centered and thus highlights the importance of accurate poverty identification to ensure the accurate assistance to the targeted poverty-stricken households [1,15]. However, according to a survey related to the challenges of conducting the targeted poverty alleviation policy, it has difficulty of accurate poverty identification and the poverty targeting mainly relies on the subjective judgment by the village committees and cadres [15]. Importantly, the difference in the regional socioeconomic level and the changing conditions may become the challenge for effective targeted poverty. When it comes to the poverty alleviation in Northeast China, the relation-based governance is prominent in rural areas, relying on relationships may occur when people want to protect their interests, which may increase the difficulty for identifying the real poor. Consequently, the difficulty comes not only from the targeted poverty alleviation policy, but also from the relation-based society. It might greatly challenge the livelihood of the poverty-stricken households. However, seemingly little attention has been paid to these challenges and the accompanying changes of the livelihood strategy of poverty-stricken households.

## 3. Literature Review

### 3.1. Sustainable Livelihoods Approach

Early research normally adopts income to measure the poverty level of households. However, a lack of income cannot be used as a useful indicator variable to fully reflect the level of deprivation of individuals or households due to its contingency [5]. To correctly measure the poverty level, a multidimensional poverty measurement index from multiple functional dimensions is necessary to be constructed. Correspondingly, the construct of feasible ability which consists of a series of functions, such as the functions of avoiding hunger and diseases, meeting the nutritional needs, receiving education, and participating in community social activities was put forward [19]. The loss of these functions is the cause of poverty, and the loss per se is the manifestation of poverty. The research based on the multidimensional poverty theory finds that, the lack of human capital is the main cause of poverty. Compared with the emphasis on increasing income, the improvement of human capital is more likely to effectively improve the feasible ability of the poor population, thus reducing the incidence of poverty [20]. However, as the multidimensional poverty theory exclusively focuses on the poor themselves but ignores the social context and institutional environment, the theory is difficult to put forward feasible policy suggestions [21].

**Figure 1:**
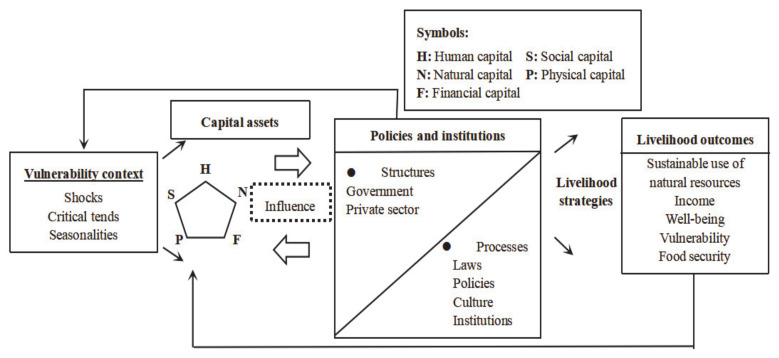
Sustainable livelihoods approach framework.

The sustainable livelihoods approach (SLA) established by the UK Department for International Development (DFID) in 2000 [22] synthesizes the previous theories [23,24,25,26], and has been adopted by many organizations and scholars [21]. Figure 1 presents the SLA framework [22]. The main part of SLA is livelihood capital assets, referring to the current situation of individuals and households. The livelihood capital assets consist of human capital, social capital and basic capital, whereas basic capital is further divided into natural capital, physical capital, and financial capital [21]. As for human capital, labour capability and education are the important important sources for human capital. As for social capital, relationships, social participation in certain groups and support from governmental institutions are the main sources. As for natural capital, for rural people, it mainly comprises agricultural lands and water. As for physical capital, public infrastructure, housing, livestock and tools for productions, appliances and other durable goods are among the important sources for livelihoods. As for financial capital, income, saving and loan are the most important sources [6]. A body of literature has investigated the evaluation of the livelihood capital. The variables such as crop planting area, water consumption, number of friends, relationship with relative, number of household electrical appliance, quality of daily diet, amount of deposit, availability of loan, health level, and employment opportunities have been seen as important indicators of livelihood capital in quantitative studies [5,6].

The choice of livelihood strategy of households highly depends on their own livelihood capital conditions [6]. It has been emphasized that livelihood strategy is sensitive to livelihood capital, in particular when the living environment is vulnerable [27]. The livelihood strategy in the SLA is a way for individuals and households to combine and use their own livelihood capital in order to achieve their livelihood goals or pursue positive livelihood output [28]. Individuals and households cope with risks and shocks through the combination of different capital assets and discover and take advantage of opportunities through the use of capital, and seek flexible conversion in various livelihood strategies to maintain their livelihood security [12,13].

Meanwhile, the SLA incorporates the vulnerability background of individuals and social structure system into the research scope [29]. As individuals or households are likely to live in a vulnerable environment and context-specific society, the comprehensive analysis of the internal capital of individuals, external policy background and social context makes the SLA more adaptive [30].

Although the correlation between a certain capital and livelihood decision-making can reflect the choice basis of individuals and households in a certain lifetime capital state, the promulgation and enforcement of the policy as well as the specific relation-based context might change the existing strategy logic and livelihoods capital assets stock of households [20]. However, the inherent logic of households’ choice has not been explored [31]. Therefore, this paper tends to explore the mechanism of households’ choice of livelihood strategy, which might be a supplement to the SLA.

### 3.2. Relation-Based Society in Rural China

Relation-based social structures dominate in most East Asian countries [32], such as China, whereas social order and society depend on complex and private relations for protection [33]. Therefore, relation-based society places an emphasis on private relationships that are closely linked to daily life [34]. In fact, relationships help to build the boundaries on “in-group” and “out-group” members or the perception of “us versus them” depending on connections and networking ties.

Contrasting to Granovetter’s weak relationship hypothesis [35], indigenous research related to the relation-based society in China proposed the strong relationship hypothesis [36]. In the relation-based society, the relationships such as kinship and geographical relations demonstrate a high possibility of favor exchanges [28], and indebtedness to the other [37] Relationships are likely to provide instrumental and emotional support for individuals and households [34].

In a relation-based society, a reliable information infrastructure is absent whereas the key information is often closed, implicit and uncodified, and the free flow of information is normally blocked. In particular, politicians are powerful and control the key information related to some policies. Thus, the access to the key information highly relies on the relationship with the key person [38]. In addition, policy-making and enforcement in a relation-based society tend to be opaque and unfair, and influenced by private relations. As a result, the information disclosure is derived from private relations, and people in relation-based society tend to circumvent the formal rules and procedures to obtain public goods and services for personal interests [39].

As people primarily rely on private ordering to obtain and protect their interests, it is common to regard private relations as an important asset and building private relationships as a necessary activity in daily life [40]. However, as it is costly to seek, evaluate each partner and maintain every relationship in a relation-based society, people prefer to engage in relationships with the ones they are most familiar with, for instance the family members, to minimize the cost. Then the second preference is to engage in relationships with friends and neighbors when their family members are powerless. The last and most reluctant preference is strangers who are most costly to search, evaluate, monitor and maintain the relationships in relation-based society [39].

When discussing poverty, poverty-stricken households are more likely to enter the vicious “poverty trap” circle in a relation-based society. It is not easy for poverty-stricken households to access to valuable information which may help them get out of poverty. Sometimes the information is available, but the information might be difficult to understand due to the lack of explanation by authoritative persons. The unfair policy enforcement might also become a major obstacle to poverty-stricken households because usually they have few private relations with powerful politicians.

In rural China, it is evident that households attach great importance to relations, in particular the relations with the clan and the neighborhood. Whenever important things happen in a household, for instance an unexpected event occurs, the clan are always the first to know and then the neighborhood [41]. Despite the importance of relationships in rural areas, how the livelihood strategy is influenced by relations in a poverty-stricken household has been seldom discussed. More importantly, at the time of the enforcement of targeted poverty alleviation policy, the existing research are mostly focused on specific poverty alleviation models [1], such as tourism poverty alleviation [42] and relocation poverty alleviation [43], how a poverty-stricken household respond to the policy when informal and private relations prevail in relation-based rural China is little known [43,44]. Therefore, the purpose of this study is to fill in the knowledge gap by borrowing the SLA to analyze households’ livelihood strategy in relation-based society facing the targeted poverty alleviation policy.

## 4. Research Design

### 4.1. Research Method

As a qualitative research method, grounded theory is able to effectively reveal the underlying reasons behind the behavior of research objects without theoretical assumptions [45]. This paper adopts the grounded theory research method with in-situ observations and in-depth interviews. Indeed a large body of literature has adopted a variety of quantitative methods to either evaluate a certain construct involved in the SLA framework, or for quantifying the relationships between these key constructs. For instance, the structural equation model was used to assess the relationship between livelihood vulnerability, livelihood capital and livelihood strategy [6]. Similarly, a sustainable livelihood analysis was utilized to quantify the SLA framework [5]. An in-depth questionnaire was widely distributed to analyse the policy innovation and challenges of targeted poverty alleviation in China [1]. Using a national survey of five provinces in the central region of China during 2015, the impact of a land use right reform on agro-environmental sustainability in rural areas was investigated through propensity score matching and a difference-in-difference model [7]. An econometric analysis of panel data at both national and regional level was conducted to examine the efficiency of an important livelihood capital, land [8]. Despite the quantitative measurement of relevant constructs and their relationship, this literature has common defects. First, data were focused on either the national or the regional level, the spatial disparity of livelihood and socioeconomic conditions has been largely neglected [5]. To offset the spatial disparity of above literature, a spatial exploratory analysis was used to measure the integrated index of livelihood [46]. Second, due to the difficulty of data collection, for instance, the most poverty-stricken households usually have a low level of education, the quality and quantity of a large-scale of questionnaire cannot be guaranteed. Thus, some indicators have been substituted or abandoned. Third, an assumption underlying these studies is the rationality of households, thus indicators have been constructed in an objective way.

Considering the defects of quantitative methods, this paper attempts to use qualitative methods to offset these defects. One of the significant advantages lie in that qualitative methods pay considerable attention to the nuances of spatial disparity, thus providing a nuanced understanding of a specific place. Meanwhile, many indicators or constructs are difficult to obtain through quantitative methods. For instance, the scope of relationships is so wide that it is difficult to quantify it, thus the quantitative data is not grounded in practice. However, it can be possible to acquire data through qualitative methods, such as narratives and story-telling. In so doing, the background key information and complex relationships can be obtained. In addition, qualitative methods can truly illustrate the imperfection of human-beings. In particular, the core of this paper belongs to the “how” question, i.e., how households make their livelihood decisions, which is of a complex and explanatory nature. Thus, instead of counting the frequency and scope of their occurrence, it is necessary to trace all kinds of interrelated events and figure out the relationship between households’ livelihood decision-making and contextual factors [47].

In short, this paper uses a grounded theory research method to analyze the relationship between relation-based society, targeted poverty alleviation and livelihood decision-making, and forms a theoretical framework of households’ livelihood decision-making under the targeted poverty alleviation in the relation-based society.

### 4.2. Case Selection

Tongyu County, which is located in the west of Jilin Province, is one of the key target counties for national poverty alleviation in Northeast China. This paper selects nineteen households in three villages of ninety poverty-stricken villages in Tongyu County as the research object. These three villages are Xinfeng Village of Xinhua Town (X Village), Bianzhao Village of Bianzhao Town (B Village) and Wujingzi Village (W Village) of Bianzhao Town. The major industries in these three villages are dominated by planting, supplemented by animal breeding. The only difference is that W Village is a relocation village. Theoretical sampling has been used to determine the number of cases, that is, when the new cases cannot provide new knowledge, the increase of cases needs to end [48]. We have contacted eighteen villages in Tongyu County and kept three villages to conduct the research. The purpose of designing multiple cases is to verify the theoretical hypothesis under different conditions and improve the accuracy and universality of the conclusion.

### 4.3. Data Source

This paper uses several sources to collect data: literature review, questionnaire, semi-structured interview and archival data, among which the primary data source is semi-structured interview. After defining the research topic, targeted poverty alleviation policy, the status of national poverty counties and SLA have been studied through literature review. In terms of questionnaire, its purpose lies in collecting quantitative data, such as age, education level, health condition and cultivated land area of the informants in order to supplement the interview data. The archives collected in this paper include the basic information of the three villages and the archives of poor households. Table 1 presents the information of the three villages.

In terms of in-depth interviews, in late July 2019, the research group went to the three poverty-stricken villages in Tongyu County, Jilin Province, to observe the rural governance and the recent situation of targeted poverty alleviation, and paid a visit to the informants for interviews. Each interview lasted about forty minutes, a total of nineteen households were interviewed. The multivariate data could provide more accurate information and obtain more robust results [49]. Table 2 presents the basic information of the informants.

The research used the interview outline to guide the interview. The interview outline involves mainly two aspects. First, the informants were asked about their understanding of the targeted poverty alleviation policy, and the degree of participation and satisfaction of the policy. Second, the relational information was collected in two parts. One part is the family information and the other part is the informants’ relationship with the village cadres and other villagers. After outlining the relationships of the informants, the informants were guided to narrate a specific event to demonstrate their relationship with the family members, village cadres and other villagers.

### 4.4. Data Analysis

Data analysis is the core of grounded theory research and is an important link between the collected data collection and generated theory. Data analysis makes theoretical propositions go beyond specific time and places to produce general interpretation capability for actions and events in different situations [49]. Data analysis could be divided into four phases, including open coding, axial coding, selective coding and theoretical framework construction [50]. The coding structure is shown in Table 3.

#### 4.4.1. Open Coding

Open codes generate directly from the original interview data. The original data are smashed up and conceptualized in a new way to achieve the purpose of clarifying phenomena, developing concepts and refining categories [48]. In this research, the data of Village W and Village B are selected for coding analysis, and the data of Village X are used for data saturation verification. Finally, eleven categories through open coding were obtained.

#### 4.4.2. Axis Coding

The task of axis coding (second-order construct) is to discover the potential logical relationship between first-order categories and develop the main category and its subcategories. Through continuous comparison, analysis and induction, this study finally forms eight main categories, including maintained type livelihood, developmental type livelihood, kinship, geographical relationship, transfusion policy, hematopoietic policy, policy quality, and livelihood capital.

#### 4.4.3. Selective Coding

Selective coding is to excavate the core dimension from the main categories, analyze the relationship between the core dimension and the main categories, and describe the behavior and phenomenon in the way of story line. Ultimately, a number of four categories were generated. A substantive theoretical framework could be developed after completing the story line.

### 4.5. Theoretical Saturation Test and Inter-Coder Reliability Test

In order to test whether the concepts and categories refined in this research have reached theoretical saturation, we conducted coding analysis on the data of Village X. We found that no new concepts and categories appeared. It indicates that the coding has reached theoretical saturation [50].

As it is important to control the data quality, the inter-coder reliability test has been conducted. Prior to the formal interview, a pilot interview was carried out. We had five interviews and transformed the recordings into transcripts for coding. After forming the coding dictionary, the theoretical logic underlying each code was carefully explained. Then the formal interviews were conducted, and two coders who are the co-authors coded the cases. The inter-coder coefficient shows a value of 0.78, indicating a reliable coding procedure [51].

## 5. Results

### 5.1. Relationships and Livelihood Strategy

The maintained type livelihood strategy refers that poverty-stricken households keep the status quo and maintain the current source of livelihood to pursue a fragile and unsustainable state of life. Among the poverty-stricken households visited by the research group, it is common that the poverty-stricken households make the maintained type livelihood due to the illness of the family members. The illness not only weakens the ability of one person to make a living, but also increases the burden of the households. In many cases, family members, especially children offer necessary helps, such as medical expenses to their old parents. B2, B3 and B6 confirmed that they received money and daily necessities from their children to maintain the life. Meanwhile, the children such as W3’s husband often stay at hometown to take care of their parents. As the informants indicated:

B2: “*I can’t do any heavy work because of illness. My son used to pay for living expenses and medical expenses.*”

W3: “*My husband gave up the opportunity to develop in the city because he had to take care of his parents in the countryside. Therefore, we make a living by doing odd jobs now.*”

In other cases, despite the good health conditions of the family members, the households still choose the maintained type of livelihood strategy. The reason is often due to the undesirable livelihood conditions, more specifically, the insufficiency of the livelihood capital. For instance, family members might stay at countryside to help each other with farming due to a lack of adequate labor force. As the informant explained:

B6:“*If he (son) leaves home, I cannot do farm work by myself. As far as I know, there are few people in our village who work outside could earn more money, so I let my son stay in the countryside to help me with the farm work.*”

Differing from the maintained type livelihood strategy, the developmental type livelihood strategy means that households change the status quo and explore new sources of livelihood through further education, skills training and production mode innovation to pursue a potentially sustainable livelihood. During the interviews, the informants delivered a strong expectation of changing their life to pursue a developmental type livelihood, but only a few of them attempted to put these plans into action. An important reason is the livelihood condition of their family members. When the livelihood conditions of their family members are desirable, these family members have the capability to support the informants’ developmental type livelihood. The possession of livelihood capital such as finance capital is a remarkable reflection of the desirable livelihood condition. As is indicated by the informants:

B7: “*This year, my son borrowed forty thousand yuan to buy sheep for me, so that I could earn some income by raising sheep.*”

W16:”*My mother’s family all know that I live a serious life, so when I went to borrow some money to start up my small business, they are glad to help and lend me money.*”

Different from the genetic relations such as family members, the geographical relations with neighborhood and villagers have a different effect on the livelihood strategy of households. In general, the neighborhood and villagers have relatively closer contact to households compared to non-neighborhood and non-villagers. When interviewing B7 whether he considered asking his neighborhood and villagers to borrow money for buying sheep, he said with a wry smile that: “all of them are poor and have no money.” But when his wife was hospitalized, he said he had borrowed money from villagers. When buying sheep, he asked favor from his son, while he asked villagers for help only in emergency. This is actually the embodiment of the aforementioned relation-based society. Considering the conditions of genetic relations themselves and other factors, B2 chose to seek help from the genetic relations to maintain life only in case of emergency.

Based on the above analysis, this paper forms the following propositions:

**Proposition** **1.** Relations have a significant effect on the choice of livelihood strategy of households. The effect of different relationships differs in the livelihood strategy of households. More specifically:

**Proposition** **1a.** When the livelihood condition of the genetic relations is undesirable, it has a high possibility to lead to the long-term maintained type livelihood strategy of households.

**Proposition** **1b.** When the livelihood condition of the genetic relations is desirable, it has a high possibility to lead to the developmental type livelihood strategy of households.

**Proposition** **1c.** When the livelihood condition of geographic relations is undesirable, it has a high possibility to lead to the short-term maintained type livelihood strategy of households.

### 5.2. Poverty Alleviation Policy and Livelihood Strategy

As for the poverty alleviation policy, it includes transfusion [52] (输血in Chinese)- type poverty alleviation policies and hematopoietic (造血in Chinese)-type poverty alleviation policies. The difference between the two lies in whether the expected effect of the policy is sustainable. Specifically, the transfusion poverty alleviation policy refers to the temporary relief of the poverty and economic tension in poverty-stricken areas by means of one-off transfer payments such as capital transfers, consumable material capital transfers and medical subsidies. The hematopoietic poverty alleviation policy refers to helping the poverty-stricken areas to develop an industrial basis for achieving a sustainable livelihood through the construction of infrastructure and public services, the transformation of industrial development modes and the transfer of productive material capital and carrying out vocational education and skill training for the poor to improve their ability to combat livelihood vulnerability and raise their living standards.

Taking the poverty alleviation subsidy of Village B (564 households) in 2018 as an example, the photovoltaic industry poverty alleviation project provided a dividend of 3000 yuan for each two- or three-star poverty-stricken households (a total of six households), and a dividend of 500 yuan for each one-star poverty-stricken households (a total of 172 households). The cooperative project of broiler breeding provided an average dividend of 365 yuan per household. For most of the poor households, the subsidy was only used for daily expenses to maintain or improve the temporary living level. The interviews indicated that no poverty-stricken households had change the maintained type livelihood strategy. For example, the informant B9 said that:

B9: “*I’m sick and take a lot of medicine. In any case, if I do some odd jobs, together with the relief fund the government deals out, I feel fine with that.*”

The transformation of the development mode belongs to the hematopoietic poverty alleviation policy, but the households have different degrees of acceptance of such policy. For example, W Village launched a sewing handicraft project. The informant W7 could not participate in the training due to the physical reason of low back pain. A similar case happened in Village B when 30 chickens were allocated to each household. Because there was no land to farm and thus no extra food for chickens, the informant B5 sold the chickens and made a profit. The above two cases show that the poverty-stricken households have no desire to change their maintained type livelihood. This might indicate that when the livelihood conditions are not satisfactory, hematopoietic poverty alleviation policies would not lead to an essential change of the poverty-stricken households’ livelihood strategy. Differently, the informant B7 decided to change her maintained type livelihood, and to try the developmental type livelihood. She kept the chickens, looked after them carefully and expected to earn some money through selling the eggs. The whole family supported her decision.

B7: “*My husband helped me build a hen house, and my son helped me mow the grass in the field to feed the chickens. My brother-in-law’s wife often came to help me feed the chickens and clean the hen house. Later eggs can produce more chickens, then I will have more eggs and earn more (money).*”

Therefore, the above discussion came to the following propositions:

**Proposition** **2.** The poverty alleviation policy has a significant effect on the livelihood strategy of households. The effect of different poverty alleviation policy differs in the livelihood strategy of households. More specifically:

**Proposition** **2a.** The transfusion alleviation poverty policy induces the maintained type livelihood strategy of poverty-stricken households.

**Proposition** **2b.** When the livelihood conditions are undesirable, the hematopoietic poverty alleviation policy induces the maintained type livelihood strategy of poverty-stricken households.

**Proposition** **2c.** When the livelihood conditions are desirable, the hematopoietic poverty alleviation policy induces the developmental type livelihood strategy of poverty-stricken households.

### 5.3. Policy Enforcement, Relationships and Livelihood Strategy

Policy-making is the prerequisite for effective enforcement of the targeted poverty alleviation policy, while the policy enforcement provides guarantee for a smooth policy-making realization. Thus, policy-making and policy enforcement are two complementary aspects related to targeted poverty alleviation policies.

Through direct observation, the research group found that poverty-stricken households’ files, the poverty alleviation records and poverty alleviation policy posters were pasted on the walls of the poor-stricken households’ houses. Meanwhile, the poverty alleviation information bulletin board was set up at the villagers’ committee. During the interviews, most of the poor households posited that poverty alleviation cadres such as the village secretary often came to visit them. The flow of policy information from the poverty alleviation department to the poor-stricken households and the livelihood information flow from the poor-stricken households to the poverty alleviation departments reflect an important part of the enforcement of targeted poverty alleviation policies.

When talking about the communication with the poverty alleviation departments, the informants pointed out that poverty alleviation cadres have the responsibility of conveying policy information, but the quality of information transmission is not satisfactory. For example, although many households have received the leaflets from the poverty alleviation cadres, they are still uncertain about the star rating of their family, subsidy payment and the progress of re-evaluation of minimum living standard. The reason is twofold. First, these informants cannot be able to understand the policy by themselves through reading the files and leaflets, and second, the poverty alleviation cadres are lacking in an effective communication with the poverty-stricken households, which reduces the possibility of these households making full use of policy conditions and achieve the goal of poverty alleviation.

The interviews also showed that when the quality of policy enforcement is unsatisfactory, the resource allocation of poverty alleviation policy highly depends on the relationships with village cadres, namely political relationships. The uneven distribution of resources due to the existence of relationships has an impact on the livelihood strategy. For instance, some informants indicated that because of the good relationship with the village cadres, it was relatively quicker to obtain the comprehensive policy information. One case is that B9 received the news about the application for the qualification of poverty-stricken households ahead of time from his cousin who is the village cadre, meanwhile, the coping strategies were explained in detail. Therefore, B9 went to the county hospital for a medical treatment certificate, so that he could make greater use of the qualification conditions to win the application. Finally, B9′s family received the corresponding policy considerations through the qualification of poverty-stricken household and could be able to manage to survive. Conversely, B6 did not get along with the village secretary due to a land dispute with a relative of the village secretary. In spite of the terrible conditions, B6′s family cannot get rated as a poverty-stricken household. As the government has no responsibility to dig wells for free for non-impoverished households, B6′s courtyard was short of water, and the green onions and other vegetables could not be sold. As B6 stated:

B6: “*We don’t have any relatives in the village cadre group. He (the village secretary) just trampled on me... And other village cadres all listen to the village secretary. Now there are college-graduate village officials. They don’t know what’s going on. They just follow the old ways. We could have made a living by selling vegetables, but now it’s really hard.*”

Therefore, the following proposition is proposed:

**Proposition** **3.** The quality of poverty alleviation policy has a significant effect on the livelihood strategy of households. More specifically:

**Proposition** **3a.** When the quality of policy enforcement is unsatisfactory, the political relationships have a high possibility to have a significant impact on the livelihood strategy of households.

## 6. Discussion

### 6.1. Theoretical Implications

Theoretically, this paper not only extends the research of poverty analysis framework, but also provides a significant perspective for enriching the long-term mechanism of targeted poverty alleviation. Specifically, first, this research incorporates the specific context of relation-based society into the poverty analysis. Taking the relation-based society as an important contextual factor, this paper explores how different types of relationships influence the livelihood strategy of households. It finds that the livelihood strategy of households highly depends on the livelihood condition of the different types of relationships. The undesirable livelihood condition of the genetic relations has a high possibility to lead to the long-term maintained type livelihood strategy of households, while the desirable livelihood condition of the genetic relations has a high possibility to lead to the developmental type livelihood strategy of households. When the livelihood condition of geographic relations is undesirable, it has a high possibility to lead to the short-term maintained type livelihood strategy of households. In so doing, this research unveils the black box of livelihood strategy in the SLA framework by embedding the strategy-making process in the specific context of relation-based society.

Second, this research explores the effect of poverty alleviation policy on affected households’ livelihood strategies. The analysis indicates that poverty alleviation policies have a significant effect on the livelihood strategy of households. More specifically, the transfusion alleviation poverty policy induces the poverty-stricken households to choose the maintained type livelihood strategy. Similarly, the hematopoietic poverty alleviation policy induces the poverty-stricken households to choose the maintained type livelihood strategy when the livelihood conditions are undesirable. By contrast, the hematopoietic poverty alleviation policy induces the poverty-stricken households to choose the developmental type livelihood strategy when the livelihood conditions are undesirable. In so doing, this research distinguishes the poverty alleviation policy into the transfusion and hematopoietic poverty alleviation policies and develops the mechanism with respect to the effect of different types of poverty alleviation policy on different types of livelihood strategy.

Third, the livelihood conditions as preconditions are introduced into the analysis of livelihood decision-making of the poverty-stricken households. This is because the households respond to policy changes in accordance with their own livelihood conditions. For example, poverty-stricken households tend to convert the allocated resources into maintenance resources to meet their current needs. The internal logic of their livelihood strategy-making helps to explain the reason why polices cannot meet the expectations in some occasions.

Last but not least, this paper explores the interaction between the quality of poverty alleviation policy, the relationships and the livelihood strategy. Specifically, the quality of poverty alleviation policy has a significant effect on the livelihood strategy of households, and the political relationships have a high possibility to have a significant impact on the livelihood strategy when the quality of policy implementation is unsatisfactory. Political relationship has been separated out as an important relationship in relation-based society. The inside logic confirms the research by Bian [36] that relationships play roles as information flow when formal institutions are absent. When the institutional environment cannot provide a sound framework or an effective enforcement of law, households tend to adopt relation-based actions to respond to the voids and protect their interests [53], among which a usual way is to establish political connections with cadres. The finding of political relationship contributes to the research of targeted alleviation policy in relation-based society from a political perspective.

### 6.2. Practical Implications

In practice, this paper provides new ideas for households to achieve sustainable livelihoods, and for governmental departments to formulate long-term poverty alleviation policies. First, it is vital to establish a sound channel of information dissemination, and to strengthen supervision and return visit mechanism. Policy information, employment information, medical information can be fully covered through online and offline information dissemination channels. In most cases, poverty is attributed to education, health condition and age, the poverty-stricken households often have poor understanding ability and have fewer channels to receive information. Therefore, both online and offline channels need to be taken seriously. Meanwhile, village-level resolutions related to poverty alleviation need to be publicized, in order to avoid internal operational risks. Second, it might be practical to carry out the expatriate system, i.e., sending grassroots cadres to another place. In this way, it might reduce the irregular operation in the process of policy implementation caused by the relationships.

### 6.3. Limitations and Future Study

With respect to the limitations and future study, first of all, the interview objects in this paper are limited to individuals, the poverty alleviation officers are not included. However, a comprehensive understanding of the role and impact of relationships in poverty alleviation policy implementation in the view of the poverty alleviation officers, such as village cadres is important. Second, this paper excludes the discussion of micro corruption. However, during the interview, the informants touched upon this issue regarding how to build relationships with village cadres. For instance, one of the informants mentioned that the minimum living allowance for his son who has a mental disease has been canceled because he did not present a gift to the village cadre. Therefore, in future studies it is critical to explore the effects of microcorruption on poverty alleviation, and further how it influences the livelihood strategy of households. Third, while livelihood capital is not a focal research topic in this paper, in the future the livelihood capital will be a focus and added to the research of poverty alleviation policy in a relation-based society. It might be interesting to investigate how the variations of livelihood capital influence the livelihood strategy under targeted poverty alleviation. Last but not least, although the qualitative method of case study was used in this paper to explore the livelihood strategy of poverty-stricken households in the relation-based society under the targeted poverty alleviation policy, it is necessary to verify the propositions proposed in this paper by using quantitative research methods, such as questionnaire survey in the future. In addition, it is critical to identify the measurement of important constructs in the conceptual framework, for instance, the measurement of livelihood capital from a multidimensional perspective.

## 7. Conclusions

Starting with the qualitative research methods of observation and interviews, this paper creatively uses grounded theory to investigate poverty-stricken households’ livelihood strategy when facing targeted poverty alleviation in a relation-based society. The paper finds that relationships have a significant impact on the poverty-stricken households’ livelihood strategy. The types of relationships have different effects on the livelihood strategies of households. This study is not only an extension of the SLA in the research context, but also provides a significant perspective for enriching the long-term mechanism of targeted poverty alleviation by building a theoretical model of the relationship between a relation-based society, targeted poverty alleviation and the livelihood strategies of households.

## Figures and Tables

**Table 1:** The basic information of the case villages.

	Xinfeng Village (X Village)	Bianzhao Village (B Village)	Wujingzi Village (W Village)
Population (household/person)	358/913	564/1328	761/2525
The poverty population filed and registered (household/person)	103/186	178/313	232/381
The real poverty population (household/person)	19/37	29/47	18/28
Average income per person 2017 (yuan)	4560	6900	3182
Average income per person 2018 (yuan)	4850	7500	3240
Village collective income 2017 (ten thousand yuan)	22	163	8
Village collective income 2018 (ten thousand yuan)	10	13.7	12
Area of cultivated land (hectare)	497	687	1212
Number of cattle/sheep	338/1696	500/2000	370/2350

**Table 3:** The Coding Structure.

Quote of Evidence	Open Coding	Axial Coding	Selective Coding
receiving daily necessities and money, receiving donation	Maintaining the status *quo*, keeping going	Maintained type livelihood	Livelihood strategy
seeking further education, learning new skills, innovating modes of production	Change the status quo and explore the new sources of livelihood	Developmental type livelihood	
children, parents, relatives	Family relations	Genetic relationship	Relationships
neighborhood, villagers	Non-family relations	Geographical relationship	
village cadres	Relationship with village cadres	Political relationship	
giving money, giving food	Transferring consumable materials	Transfusion policy	Poverty alleviation policy
amending roads	Building infrastructure, training skills, production material support	Hematopoietic policy	
efficiency of information flow, standardization of policy implementation	Information transparency, implementation normalization	Policy quality	
Health issue, education	Human capital	Livelihood capital	Livelihood condition
tools, livestock	Physical capital		
pension, living expenses	Finance capital		
land, climate, weather	Natural capital		

**Table 2:** Basic information of informants.

ID	Poverty Level	Gender	Age	Health Condition	Education Level
B-2	★★★	Male	>60	Sick and able to participate in a little work	Primary
B-3	★★	Male	>60	Sick and able to participate in a little work	Secondary
B-4	★	Female	>60	Sick and able to participate in moderate work	Primary
B-5	★	Female	>60	Sick and unable to work	Secondary
B-6		Female	>60	Sick and able to participate in moderate work	Primary
B-7		Female	<60	In good condition	Primary
B-9	★	Male	>60	Sick and able to participate in a little work	Primary
W-3	★★★	Female	<60	In good condition	Primary
W-7	★	Female	>60	Sick and able to participate in a little work	Secondary
W-10		Female	<60	In good condition	Secondary
W-16		Female	<60	In good condition	Secondary
X-1	★	Female	>60	In good condition	No
X-2	★	Female	>60	In good condition	Secondary
X-3	★★★	Female	>60	In good condition	Primary
X-4	★	Male	>60	Sick and able to participate in a little work	Primary
X-5	★★	Male	>60	In good condition	No
X-6	★★★	Male	>60	Sick and unable to work	Primary
X-7		Male	<60	Sick and unable to work	Secondary
X-8		Female	<60	In good condition	Primary

Note: For the first column “ID”, B, W, and X represent Bianzhao Village, Wujingzi Village and Xinfeng Village, respectively, and the number represents the serial number of informant. For the second column “Poverty Level”, ★, ★★, ★★★ represent general poverty, moderate poverty and extreme poverty, respectively.

## Data Availability

Data is available upon request by contacting the corresponding author.
